# Mesh fixation techniques for inguinal hernia repair: an overview of systematic reviews of randomised controlled trials

**DOI:** 10.1007/s10029-021-02546-x

**Published:** 2021-12-14

**Authors:** A. Alabi, N. Haladu, N. W. Scott, M. Imamura, I. Ahmed, G. Ramsay, M. Brazzelli

**Affiliations:** 1 Institute of Applied Health Sciences, University of Aberdeen, Aberdeen, UK; 2 Luton and Dunstable University Hospital, Luton, UK; 3 Emergency Department, Southend University Teaching Hospital, Westcliff-on-Sea, UK; 4 Medical Statistics Team, University of Aberdeen, Aberdeen, UK; 5 Health Services Research Unit, University of Aberdeen, Aberdeen, UK; 6 Department of Surgery, NHS Grampian, Aberdeen, UK

**Keywords:** Hernia repair, Inguinal, Mesh, Overview of systematic reviews

## Abstract

**Purpose:**

Inguinal hernia repair using surgical mesh is a very common surgical operation. Currently, there is no consensus on the best technique for mesh fixation. We conducted an overview of existing systematic reviews (SRs) of randomised controlled trials to compare the risk of chronic pain and recurrence following open and laparoscopic inguinal hernia repairs using various mesh fixation techniques.

**Methods:**

We searched major electronic databases in April 2020 and assessed the methodological quality of identified reviews using the AMSTAR-2 tool.

**Results:**

We identified 20 SRs of variable quality assessing suture, self-gripping, glue, and mechanical fixation. Across reviews, the risk of chronic pain after open mesh repair was lower with glue fixation than with suture and comparable between self-gripping and suture. Incidence of chronic pain was lower with glue fixation than with mechanical fixation in laparoscopic repairs. There were no significant differences in recurrence rates between fixation techniques in open and laparoscopic mesh repairs, although fewer recurrences were reported with suture. Many reviews reported wide confidence intervals around summary estimates. Despite no clear evidence of differences among techniques, two network meta-analyses (one assessing open repairs and one laparoscopic repairs) ranked glue fixation as the best treatment for reducing pain and suture for reducing the risk of recurrence.

**Conclusion:**

Glue fixation may be effective in reducing the incidence of chronic pain without increasing the risk of recurrence. Future research should consider both the effectiveness and cost-effectiveness of fixation techniques alongside the type of mesh and the size and location of the hernia defect.

## Introduction

Abdominal wall hernia is a common clinical manifestation in general surgery, with a prevalence of 4% for people over 45 years of age and 1.7% for all ages. [1] Seventy-five percent of abdominal wall hernias are inguinal hernias. The lifetime risk of inguinal hernia repair is 27% in men and 3 in women [2]. While most inguinal hernias have a slow natural course and result in mild to moderate discomfort. However, occasionally they may cause severe complications such as bowel obstruction or strangulation.

Inguinal hernia repair ranks among the most performed surgical procedure in general surgery, with up to 80,000 procedures per year in the UK alone, and over 20 million procedures worldwide [3, 4]. Up to 15% of these repairs are for recurrences having previously had an attempted repair [5].

Today, the treatment of choice is hernia repair involving the placement of a mesh performed either through open or laparoscopic surgery. The use of mesh varies worldwide from 0 to 5% in low-resource countries to 95% in high-resource countries [6]. Mesh repairs offer established advantages such as lower incidence of chronic groin pain, faster convalescence, and fewer hernia recurrences [7, 8]. In current clinical practice, open approaches involving the placement of a mesh include flat mesh (e.g., Lichtenstein repair) pre-peritoneal repair, and the plug-and-mesh system. Laparoscopic approaches include the totally extraperitoneal (TEP) and the transabdominal preperitoneal (TAPP) repairs.

The incidence of moderate to severe chronic groin pain following inguinal mesh repair is reported to be around 10%–12% [9, 10] It is attributed to multiple factors, including nerve injury or entrapment during fixation. Specifically, mesh-related factors such as mesh material, mesh pore size and the mesh fixation method are considered to play an important role in the development of groin pain. Similarly, mesh properties such as inertness, resistance to infection, ability to retain tensile strength long-term, absorption into host tissue may influence the likelihood of recurrence [11].

Several randomised controlled trials (RCTs) and systematic reviews have compared various mesh fixation methods in both open and laparoscopic surgical procedures to assess clinical outcomes such as recurrence, post-operative infection, the incidence of chronic groin pain, and time-to-recovery. However, overall evidence in support of any specific fixation method has been conflicting. Furthermore, no attempt has been made to summarise the body of evidence from published systematic reviews [12–18]. Identification of optimal fixation methods for inguinal hernia repairs would enhance surgical care and the quality of life of potentially millions of patients globally. According to the UK Hospital Episode Statistics, in England for 2019–2020 the number of recorded hernia repairs was 64,769. Of these procedures, 59,664 were for the repair of primary hernias and 5105 for the repair of recurrent hernias [19]. Any reduction in the number of surgical operations would translate into significant reductions in resource use and costs for any healthcare system in the world.

Overviews of systematic reviews are a relatively novel methodology, which offers the advantage to summarise different interventions for the same condition or clinical population where several systematic reviews already exist [20]. Considering the number of existing reviews in this clinical area, to inform clinical practice and prevent the need for further systematic reviews we decided to perform an overview of existing systematic reviews of randomised controlled trials, to collect, analyse and present data on inguinal hernia repairs using mesh. Incidence of chronic pain and recurrence rate were selected as primary outcomes.

## Methods

This overview aimed to summarise the evidence from existing published systematic reviews on the benefits and risks of mesh fixation techniques for open and laparoscopic inguinal hernia repairs in adults.

### Study design and research protocol

The methods of this overview were pre-specified in a research protocol according to the recommendations outlined in the Cochrane Handbook of Systematic Reviews of Interventions [21] and the PRISMA statement [22].

### Eligibility criteria

We included published systematic reviews of randomised controlled trials (RCTs), which focused on mesh fixation techniques for the repair of inguinal hernia and met the following inclusion criteria:

- Study designs: systematic reviews and/or meta-analyses of RCTs.
- Participants: adults with unilateral or bilateral inguinal hernia who underwent an open or laparoscopic mesh repair;
- Intervention: methods for mesh fixation (including but not limited to self-gripping, suture, glue, staple, or tack fixation);
- Comparator: any comparator intervention investigated;
- Outcome measures: chronic groin pain and recurrence.
- Where a systematic review included both RCTs and non-RCTs, we included and analysed only the subset of randomised studies.

### Literature search

To identify eligible reviews published in English, we searched major electronic databases, (i.e., MEDLINE, EMBASE, and Cochrane Database of Systematic Reviews) in April 2020. We used both MeSH and text search terms (i.e., inguinal hernia, groin hernia, femoral hernia, surgery, open hernia, laparoscopic repair, endoscopic repair, total extraperitoneal repair, transabdominal preperitoneal repair, TAPP, TEP, self-gripping, suture, glue, tack, staple, systematic review, meta-analysis) and combined them appropriately with the Boolean connectors, “AND” and “OR”. We did not apply any restriction in terms of publication date. In addition, we perused the reference lists of identified systematic reviews for any further relevant references.

### Data extraction and quality assessment of included reviews

Two researchers (AA and NH) independently screened the results of the searches and selected articles based on their title and abstract with disagreement being resolved by discussion or consultation with a third reviewer (screening phase). Articles that appeared to be potentially relevant were retrieved in full and analysed by the same two reviewers in line with the pre-specified inclusion criteria. For each included review, data were extracted by one author (AA) using a form developed and piloted for this overview. Information on the following items was recorded: administrative details and scope of the review, review characteristics, number of RCTs, characteristics and number of participants, characteristics of interventions and comparators, outcome measures (recurrence and chronic groin pain), and review results. For systematic reviews that included a meta-analysis (MA) or a network meta-analysis (NMA), quantitative effect measures, including confidence intervals (CI) or credible Intervals (CrI), were recorded.

The quality of the included systematic reviews was assessed by one reviewer using the validated AMSTAR-2 (A Measurement Tool to Assess systematic Reviews-2), which comprises 16 items. [23] As well as assessing each item separately, the authors of AMSTAR-2 recommend deriving an overall assessment based on the presence of factors deemed critically important. In line with their recommendations, we made appropriate modifications to tailor the tool to this overview. The following items were regarded as critical: adequacy of the literature search; justification for excluding individual studies; appropriateness of meta-analytical methods; and assessment of risk of bias of included reviews and its consideration in interpreting the review results. For each review, we provided a rating of the overall confidence in the results of the review. We did not exclude reviews based on AMSTAR-2 ratings but considered the ratings in the interpretation of our results.

### Data synthesis

We summarised the main characteristic of each review (number of included studies, number of included participants, date of literature search, inclusion criteria, type of mesh fixation technique, and duration of follow up) and synthesised data on outcomes of interest (chronic pain and recurrence rate) using summary tables [20]. We did not attempt to standardise numerical results across reviews, as data on comparative effectiveness and information on adverse events were heterogeneously measured and reported. Reviews that included evidence from both RCTs and non-RCTs were only included in the data synthesis if the results of the RCTs were presented separately as a distinct subgroup.

## Results

### Literature search

**Fig. 1:**
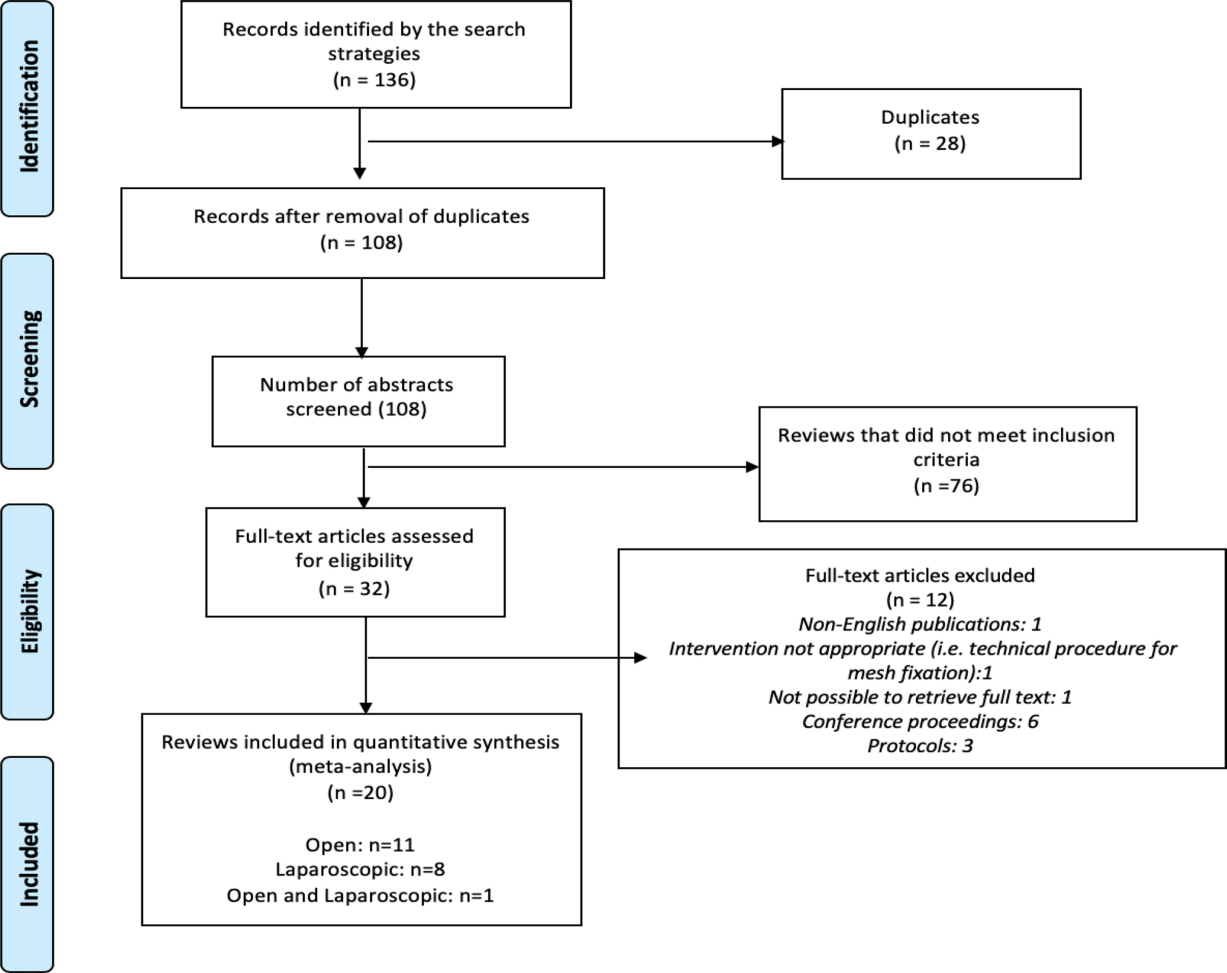
PRISMA flow diagram

The initial search retrieved 136 records. After the removal of duplicates, 108 abstracts were screened for eligibility. After full-text assessment of 32 potentially relevant articles, 20 systematic reviews (with or without meta-analyses) met the inclusion criteria and were included in the overview. Twelve reviews were excluded as they did not meet the pre-specified inclusion criteria. The main reason for exclusion were non-English publications (*n* = 1), full-text articles not available (i.e., conference proceedings; study protocols) (*n* = 9), no access to the full-text article (*n* = 1). The PRISMA flow chart summarising the study selection process is shown in Fig. 1.

### Study details

The characteristics of the 20 included reviews are summarised in Table 1. The included reviews were published between 2011 and 2019. The number of RCTs per review varied from 1 to 28, and the number of study participants ranged from 93 to 5190. All reviews included both male and female participants with a predominance of men; age of participants ranged from 17 to 86 years across reviews.

**Table 1:** Characteristics of the systematic reviews and meta-analyses included in the overview

Review ID	Search dates	Number of RCTs	Number of participants	Age, years (range of reported mean or median)	Sex (All/ M/F)	Type of hernia		Type of surgical technique		Notes
						Primary or recurrent	Unilateral or bilateral	Open or laparoscopic approach	Fixation techniques compared	
Open surgery										
De Goede [32]	Jan 1990 -April 2012	7	1185	52.3–63	All	NR	UH	Open (Lichtenstein)	Glue vs. Suture	
Ismail 2017 [27]	July 2016	12	3483	38.1–66.8	All	NR	UH, BH	Open	Self-gripping vs. Suture	
Ladwa [16]	Feb 2012	7	1259	49–60.5	All	NR	UH, BH	Open	Glue vs. Suture	
Lin [26]	July 2017	13	2375	44.2—60.6	NR	PH	NR	Open	Glue vs. Suture	
Liu [29]	August 2013	4	585	NR	NR	NR	UH, BH	Open	Glue vs. Suture	Results of RCTs not reported separately from those of non-RCTs
Rausa [24]	May 2018	28	5495	> 18	All	PH, RH	UH, BH	Open	Glue vs Suture vs. Self-gripping	NMA
Sajid [31]	NR	4	1115	49–60	All	PH	UH	Open	Self-gripping vs. Suture	
Sanders [33]	August 2012	12	1992	> 18	NR	NR	NR	Open	Suture vs. Glue vs Self-gripping vs. Tack	Narrative summary
Sun [9]	May 2016	12	1932	> 18	All	PH	UH, BH	Open (Lichtenstein)	Glue vs Suture	
Van Steensel [25]	May 2017	23	5190	44–67	NR	PH	UH	Open	Suture vs. Glue; Suture vs. Self-gripping	
Zhang [34]	Jan 2005—Feb 2013	4	1353	49–66.8	NR			Open	Self-gripping vs. Suture	Results from of RCTs not reported separately from those of non-RCTs
Laparoscopic surgery										
Antoniou [36]	Jan 2015	9	1454	> 18	All	PH, RH	UH, BH	Lap (TAPP or TEP)	Glue vs Mechanical fixation (tack, strap, staple)	
Kaul [18]	Dec 2010	1	93	Staple 66; Glue 64 (Mean)	Staple 47/0; Glue 45/1 (M/F)	PH, RH	BH	Lap (TEP)	Glue vs. Staple	
Lederhuber [17]	Jan 1990—June 2015	14	2161	42.5–59.6	NR	PH, RH	UH, BH	Lap (TAPP or TEP)	Any mesh fixation techniques (including no fixation)	Narrative Summary
Li [37]	Oct 2013	8	1228		NR			Lap	Glue vs. Staple	
Sajid [30]	Sep 2011	5	1001	42–66	All	NR	UH, BH	Lap (TAPP or TEP)	Mechanical fixation (tack, staple) vs. Glue	
Shah [15]	Jan 1990—June 2013	5	526	49–66	All	NR	UH, BH	Lap (TAPP or TEP)	Tack vs Glue	
Shi [14]	Feb 2016	4	430	45.5–57.7	NR	PH, RH	UH, BH	Lap (TAPP)	Staple vs Glue	
Techapongsatorn [35]	Feb 2018	15	1783	27.3–65.8	All	PH, RH	UH, BH	Lap (TEP)	Tack vs Suture, Glue, Self-gripping mesh and no fixation	NMA
Open and laparoscopic surgery										
Fortelny [38]	NR	8	1556	NR	NR	NR	NR; in one RCT patient acted as own control (bilateral)	Open and lap	Fibrin sealant vs any other	Narrative Summary

*BH* bilateral hernia; *Lap* laparoscopic; *M/F* Male/Female; *NMA* network meta-analysis; *NR* not reported; *RCT* randomised controlled trial; *TAPP* transabdominal preperitoneal approach; *TEP* totally extraperitoneal approach; *UH* unilateral hernia

Eleven of the included reviews summarised the evidence of different mesh fixation techniques in open inguinal hernia repairs, [16, 24–29, 31–34]; eight reported mesh fixation in laparoscopic inguinal hernia repairs [14, 15, 17, 18, 30, 35–37] and one review reported mesh fixation in both open and laparoscopic repairs [38].

Open fixation techniques considered in the included reviews were glue, suture, and self-gripping mesh. Laparoscopic fixation techniques included glue, staple, suture, and tack. Techapongsatorn et al. split tacks into metallic and absorbable tacks and analysed these separately [35]. Antoniou et al. combined tacks, secure straps, staples, or ‘*any other permanent or absorbable material penetrating the abdominal wall to secure the mesh’* as ‘mechanical fixation’ [36]. Sajid et al., studied staples and tacker fixation together and grouped them under ‘mechanical fixation’ [30].

The main characteristics of the included systematic reviews are reported in Table 1.

### Methodological quality of included systematic reviews

One review was judged to be of high quality, [9] and there were 10 reviews of moderate quality, [14, 17, 24–26, 29, 33, 35–37] 5 reviews of low quality [15, 27, 30, 32, 34] and 4 reviews of critically low quality (Table 2) [16, 18, 31, 38].

**Table 2:** AMSTAR-2 quality assessment summary

Review ID	1	2	3	4	5	6	7	8	9	10	11	12	13	14	15	16	Overall confidence rating
Antoniou [36]	Y	Y	N	Y	Y	Y	Y	Y	Y	Y	Y	Y	N	Y	Y	Y	Moderate
De Goede [32]	Y	N	N	Y	Y	Y	Y	Y	Y	N	Y	Y	Y	N	Y	Y	Low
Fortelny [38]	N	N	N	N	N	N	N	PY	N	N	NMC	NMC	N	N	NMC	Y	Critically Low
Ismail [27]	Y	Y	N	Y	Y	Y	N	Y	Y	N	Y	Y	Y	Y	Y	N	Low
Kaul [18]	Y	N	N	N	Y	N	N	Y	N	N	Y	Y	Y	N	N	Y	Critically Low
Ladwa [16]	N	N	N	Y	Y	Y	Y	Y	N	N	Y	N	N	Y	N	Y	Critically Low
Lederhuber [17]	Y	Y	N	Y	Y	N	Y	PY	Y	N	NMC	NMC	Y	Y	NMC	Y	Moderate
Li [37]	Y	N	N	Y	Y	Y	Y	PY	Y	N	Y	Y	Y	Y	Y	N	Moderate
Lin [26]	Y	N	N	Y	N	Y	Y	Y	Y	N	Y	N	Y	N	N	Y	Moderate
Liu [29]	N	N	Y	Y	N	Y	PY	PY	Y	N	Y	Y	Y	Y	Y	Y	Moderate
Rausa [24]	Y	PY	N	Y	N	Y	Y	N	Y	N	Y	N	N	N	N	N	Moderate
Sajid [30]	N	N	N	Y	Y	Y	Y	Y	Y	N	N	N	Y	Y	N	Y	Low
Sajid [31]	N	N	N	N	N	Y	Y	Y	PY	N	N	N	N	N	N	Y	Critically Low
Sanders [33]	Y	N	N	Y	Y	N	Y	PY	PY	N	NMC	NMC	Y	Y	NMC	Y	Moderate
Shah [15]	N	N	N	Y	N	N	N	Y	Y	N	N	Y	N	Y	Y	Y	Low
Shi [14]	N	N	N	Y	N	N	PY	Y	Y	N	Y	Y	Y	N	N	Y	Moderate
Sun [9]	Y	Y	N	Y	Y	Y	Y	Y	Y	Y	Y	Y	Y	Y	Y	Y	High
Techapongsatorn [35]	Y	Y	N	Y	Y	Y	Y	PY	Y	N	Y	N	N	Y	Y	Y	Moderate
Van Steensel [25]	Y	Y	N	Y	Y	N	Y	Y	Y	N	Y	N	Y	Y	Y	Y	Moderate
Zhang [34]	Y	PY	Y	Y	N	Y	Y	PY	Y	N	N	N	N	Y	N	N	Low

Key: 1- PICO in research question; 2- Pre-established methodology and protocol; 3- Study design justified; 4- Comprehensive literature search; 5- Duplicate study selection; 6- Duplicate data extraction; 7- Excluded studies listed & justified; 8- Included studies well-described; 9- Risk of bias appropriately assessed; 10- Funding sources of included studies highlighted; 11- Appropriate statistical analysis; 12- RoB impact assessed; 13- Impact of ROB on interpretation of results discussed; 14- Heterogeneity investigated and impact discussed; 15- Publication bias; 16- Conflicts of interest; *Y* Yes; *N* No; *PY* Partial Yes; *NMC* No meta-analysis conducted

### Synthesis of Results

#### Chronic groin pain

All identified systematic reviews assessed ‘chronic pain’ and some provided results for both acute/early postoperative pain and chronic pain. However, definitions and measurements of chronic pain varied across reviews. Most reviews assessed chronic pain at or ‘beyond 3 months’ after surgery, some reported chronic pain at 12 months, and one review up to 5 years. Table 3 presents the results for chronic groin pain for both open and laparoscopic mesh repairs.

**Table 3:** Summary of results for chronic pain after open and laparoscopic inguinal hernia repairs across systematic reviews

Review ID	Outcome definition (time from surgery)*	RCTs (no of participants)	Chronic pain rates	Effect Measure	Effect Size (95% CI/Crl)	AMSTAR-2 judgement	Comments
Open mesh repair							
Glue versus Suture (OR, RR > 1 favour suture)							
Van Steensel [25]	12 months	9 (1981)	Glue 68/991 (6.9%) Suture 166/990 (16.8%)	OR	0.43 (0.11, 1.74)	M	
Lin [26]	Early: 3 months	9 (1718)	Glue 62/860 (7.2%) Suture 91/858 (10.6%)	OR	0.58 (0.32, 1.03)	M	
	Late: 5 years	2 (566)	Glue 16/282 (5.7%) Suture 25/284 (8.8%)	OR	0.62 (0.32, 1.19)	M	
Sun [9]	Early: 3 months	10 (1473)	Glue 52/732 (7.1%) Suture 80/741 (10.8%)	OR	**0.63 (0.44, 0.91)**	H	
De Goede [32]	Early: 3–6 months	4 (772)	Glue 22/385 (5.7%) Suture 48/387 (12.4%)	RR	**0.52 (0.31, 0.87)**	L	
	Late: 12 months	3 (852)	Glue 58/423 (13.7%) Suture 65/429 (15.1%)	RR	0.88 (0.54, 1.42)	L	
Ladwa [16]	NR	5 (1157)	Glue 55/601 (9.2%) Suture 73/556 (13.1%)	RR	0.63 (0.30, 1.28)	CL	
Sanders [33]	3–12 months	8 (1336)	Glue 57/667 (8.5%) Suture 81/669 (12.1%)	NA	NA	M	No meta-analysis was conducted. Three RCTs reported a significant reduction of pain with glue compared with suture, while 5 RCTs reported no significant differences
Glue versus Self-gripping (RR > 1 favours self-gripping)							
Rausa [26]	12 months	20 (NR)	NR	RR	0.63 (0.36, 1.12)	M	NMA conducted
Self-gripping versus Suture (RR, OR > 1 favour suture)							
Rausa [24]	12 months	20 (NR)	NR	RR	0.91 (0.63, 1.45)	M	NMA conducted
Van Steensel [25]	12 months	6 (1498)	Self-gripping 50/738 (6.8%) Suture 36/760 (4.7%)	OR	1.45 (0.92, 2.28)	M	
Ismail [27]	3 months	7 (1417)	NR	OR	0.82 (0.58, 1.18)	L	
Sajid [31]	NR	3 (1025)	Self-gripping 75/508 (14.8%) Suture 73/517 (14.1%)	OR	1.04 (0.72, 1.49)	CL	
Sanders [33]	3–12 months	2 (408)	Self-gripping (18.2%) Suture (14.7%)	NA	NA	M	No meta-analysis was conducted. No significant differences between techniques were reported
Mechanical fixation (tack) versus suture							
Sanders [33]	3–12 months	1 (34)	0% in each group	NR	NR	M	No significant differences between techniques were reported
Laparoscopic mesh repair							
Glue versus Mechanical fixation (RR, OR > 1 or RD > 0 favour mechanical fixation [tack and/or staple])							
Antoniou [36]	> 3 months	4 (454)	Glue 14/226 (6.2%) Mechanical fixation 27/228 (11.8%)	OR	**0.46 (0.22, 0.93)**	M	
Sajid [30]	NR	4 (912)	Glue 5/306 (1.6%) Mechanical fixation 43/606 (7.0%)	RR	**0.22 (0.09, 0.54)**	L	
Shah [15]	3 months	4 (491)	Glue 13/244 (5.3%) Mechanical fixation 31/247 (12.6%)	Peto OR	**0.40 (0.21, 0.76)**	L	
Shi [14]	> 1 month	4 (1558)	Glue 37/704 (5.3%) Mechanical fixation 54/854 (6.3%)	NR	NR	M	No meta-analysis was conducted. Two RCTs reported no differences between the two groups, while 2 found a statistically significant lower pain score in the fibrin glue group
Li [37]	3 months	6 (1039)	Glue 16/368 (4.3%) Mechanical fixation 56/671 (8.3%)	RD	**−0.06 (−0.08, −0.04)**	M	
Kaul [18]	12 months	1 (93)	Glue (13.2%) Mechanical fixation (20.0%)	NA	NA	CL	
Techapongsatorn [35]	NR	11 (1496)	NR	RR	0.53 (0.25, 1.12)	M	NMA conducted
Glue versus Suture (RR > 1 favour suture)							
Techapongsatorn [35]	NR	11 (1496)	NR	RR	0.20 (0.01, 4.47)	M	NMA conducted
Self-gripping versus Glue (OR, RR > 1 favour glue)							
Lederhuber [17]	> 3 months	1 (100)	Self-gripping 0/47 (0%) Glue 0/49 (0%)	NR	NR	M	
Suture versus Mechanical fixation (RR > 1 favour mechanical fixation [metallic tack])							
Techapongsatorn [35]	NR	11 (1496)	NR	RR	2.58 (0.11, 61.71)	M	NMA conducted

*NA* = not applicable; *NMA* = network meta-analysis; *NR* = not reported; *OR* = odds ratio; *RR* = risk ratio; *RD* = risk difference; *H* = High, *M* = Moderate, *L* = Low, *CL* = Critically low; *RCT* = randomised controlled trial

*3 months could mean ‘at’ or 'beyond (at least)' 3 months

Results in bold indicate a significant difference between treatment groups

For open mesh repairs, six systematic reviews compared glue fixation with suture. [16, 25, 26, 28, 32, 33] and five of them included a meta-analysis. [16, 25, 26, 28, 32] Meta-analysis results across reviews tended to indicate that glue fixation has a lower risk of chronic pain compared with suture, and two systematic reviews, one at low risk of bias [28] and one at high risk of bias, [32] reported statistically significant differences in the short-term [3–6 months after surgery] [OR 0.63; 95% CI 0.44 to 0.91 and RR 0.52; 95% CI 0.31 to 0.87, respectively]. One review of moderate quality [26] that assessed chronic pain at 5 years did not find a statistically significant difference between glue and suture fixation in the long-term (OR 0.62; 95% CI 0.32 to 1.19).

Five systematic reviews compared self-gripping mesh with suture and found no significant differences in chronic pain between the two fixation techniques. [24, 25, 27, 31, 33] Similarly, one review comparing self-gripping mesh with glue [24] and another review comparing mechanical fixation (tack) with suture fixation for open mesh repair [33] reported comparable rates of chronic pain between fixation techniques. However, most of these reviews reported wide confidence intervals (CIs) around the summary estimate of effect, and clinically important effects favouring either technique for mesh fixation cannot be ruled out with certainty (Table 3).

Regarding laparoscopic mesh fixation techniques, 6 systematic reviews assessed glue fixation versus mechanical fixation (tack and/or staple) and 4 of these combined the results of the included RCTs in a meta-analysis. Results of these reviews were consistent in showing a lower incidence of chronic pain after glue fixation than after mechanical fixation, although effect sizes varied between reviews (Antoniou et al.  = OR 0.46; 95% CI 0.22 to 0.93; Sajid et al.  = RR 0.22; 95% CI 0.09 to 0.54, Shah et al.  = OR 0.40; 95% CI 0.21 to 0.76; Li 2015 = RD − 0.06; 95% CI − 0.08 to − 0.04). [15, 30, 36, 37] On the other hand, a network meta-analysis published in 2018 did not find any statistically significant difference in rate of chronic pain between glue fixation and mechanical fixation using metallic tack (RR 0.53, 95% CI 0.25 to 1.12); however, the ranking of interventions favoured glue, which was reported to have a 47% lower risk of chronic pain than mechanical fixation [35]. The same network meta-analyses did not show evidence of a difference between glue fixation and suture, between suture and mechanical fixation using metallic tack, or between mechanical fixation using metallic tack and no fixation. However, glue fixation was ranked the best for lowering the incidence of chronic pain compared with suture. In addition, suture showed a higher risk of chronic pain than mechanical fixation [35]. It is worth noting that most reviews did not report results for TAPP and TEP approaches separately.

#### Hernia recurrence

Table 4 presents the results for hernia recurrence across the included systematic reviews. The timeframe for assessing recurrence was not clearly reported in most of the identified systematic reviews. Where reported, the incidence of recurrence was assessed after a follow-up period of 6 to 12 months. Six systematic reviews assessed glue fixation versus suture for open inguinal repair; 4 of these reviews performed a meta-analysis. While there was a trend in favour of suture (fewer recurrences), no significant differences were observed between the two fixation techniques across reviews (Table 4). Five systematic reviews comparing self-gripping mesh versus suture in open inguinal repair reported similar rates of recurrence between fixation techniques. A network meta-analysis of moderate quality published in 2019 showed that the risk of recurrence was similar between glue fixation and self-gripping mesh. Seven systematic reviews of variable quality comparing glue fixation with mechanical fixation (tack or staple) for laparoscopic inguinal repair showed no significant differences between fixation techniques. Similarly, a network meta-analysis of moderate quality published in 2018 reported no significant differences among various methods of mesh fixation using a laparoscopic approach but ranked glue fixation and suture as the best options for lowering recurrence (approximately 71% lower risk) and mechanical fixation using absorbable tack as the worst [35]. It is worth noting that most of the meta-analyses included in the identified systematic reviews showed wide confidence intervals and, therefore, clinically important effects could not be ruled out with certainty.

**Table 4:** Summary of results for hernia recurrence after open and laparoscopic inguinal hernia repairs across systematic reviews

Review name	Outcome definition (time after procedure)	RCTs (no of participants)	Recurrence rates	Effect Measure	Effect Size (Confidence /Credible Interval)	AMSTAR-2 judgement	Comments
*Open mesh repair*							
Open repair: Glue versus Suture (OR or RR > 1 favours suture)							
Van Steensel [25]	NR	NR	NR	OR	Fibrin glue 1.34 (0.25, 7.07) Cyanoacrylate glue 1.53 (0.48, 4.86)	M	No significant differences in recurrence rates in separate comparisons of glue *versus* suture fixation were reported
Lin [26]	Early: 12 months	11 (2265)	Glue 7/1114 (0.6%) Suture 5/1151 (0.4%)	OR	1.32 (0.47, 3.69)	M	
	Late: 60 months	2 (566)	Glue 12/282 (4.3%) Suture 8/284 (2.8%)	OR	1.54 (0.62, 3.83)	M	
Sun [9]	NR	12 (1987)	Glue 13/970 (1.3%) Suture 9/1017 (0.9%)	OR	1.44 (0.63, 3.28)	H	
De Goede [32]	NR	6 (1003)	Glue 11/495 (2.2%) Suture 10/508 (1.9%)	RR	1.26 (0.54, 2.92)	L	
Ladwa [16]	NR	5 (1203)	NR	RR	1.23 (0.52, 2.94)	CL	
Sanders [33]	NR	8 (1227)	Glue 12/606 (1.9%) Suture 11/621 (1.8%)	NA	NA	M	No meta-analysis was conducted. No significant differences were reported
Self-gripping versus Suture (OR or RR > 1 favours suture)							
Rausa [24]	12 months	NR	NR	RR	1.54 (0.83, 2.78)	M	NMA conducted
Van Steensel [25]	NR	NR	NR	OR	0.98 (0.52, 1.86)	M	
Ismail [27]	NR	7 (2289)	NR	OR	1.13 (0.5, 2.23)	L	
Sajid [31]	NR	4 (1115)	Glue 2/553 (0.4%) Suture 3/562 (0.5%)	OR	0.76 (0.14, 4.08)	CL	
Sanders [33]	NR	2 (444)	Self-gripping 0/222 (0%) Suture 1/222 (0.5%)	NA	NA	M	No significant differences were reported
Glue versus Self-gripping (OR or RR > 1 favours self-gripping)							
Rausa [24]	12 months	NR	NR	RR	1.5 (0.52, 4.70)	M	NMA conducted
Open repair: Tack versus Suture (OR or RR > 1 favours suture)							
Sanders [33]	NR	1 (34)	0% in each group	NA	NA	M	No significant differences were reported
*Laparoscopic mesh repair*							
Glue versus Mechanical fixation (OR or RR > 1 and RD > 0 favours mechanical fixation)							
Antoniou [36]	12 months (median)	8 (841)	Glue 6/419 (1.4%) Mechanical fixation 4/422 (0.9%)	OR	1.45 (0.45, 4.55)	M	
Sajid [30]	NR	5 (1001)	Glue 5/350 (1.4%) Mechanical fixation 6/651 (0.9%)	RR	1.19 (0.39, 3.70)	L	
Shi [14]	NR	4 (430)	Glue 7/215 (3.3%) Mechanical fixation 3/215 (1.4%)	OR	2.10 (0.61, 7.22)	M	
Li [37]	6 months	8 (1228)	Glue 9/462 (1.9%) Mechanical fixation 8/766 (1.0%)	RD	-0.00 (-0.01, 0.01)	M	
Kaul [18]	NR	1 (93)	0% in each group	NA	NA	CL	
Shah [15]	NR	5 (523)	Glue 7/271 (2.6%) Mechanical fixation 3/252 (1.2%)	Peto OR	2.36 (0.67, 8.37)	L	
Techapongsatorn [35] (glue versus metallic tack)	NR	15 (1829)	NR	RR	0.29 (0.07,1.30)	M	NMA conducted
Techapongsatorn [35] (glue versus absorbable tack)	NR	15 (1829)	NR	RR	0.04 (0.00, 2.74)	M	NMA conducted
Glue versus Suture (RR > 1 favours suture)							
Techapongsatorn [35]	NR	15 (1829)	NR	RR	1.02 (0.02, 51.21)	M	NMA conducted
Suture versus Mechanical fixation (RR > 1 favours mechanical fixation)							
Techapongsatorn [35] (suture versus metallic tack)	NR	15 (1829)	NR	RR	0.29 (0.00, 18.81)	M	NMA conducted
Techapongsatorn [35] (suture versus absorbable tack)	NR	15 (1829)	NR	RR	0.04 (0.00, 12.40)	M	NMA conducted
Lederhuber [17] (suture versus staple)	12 months	1 (236)	Suture 1/120 (0.8%) Mechanical fixation 0/116 (0%)	NR	NR		No significant differences were reported. Data from this trial were not clearly reported in the systematic review
Absorbable tack versus Metallic tack (RR > 1 favours metallic tack)							
Techapongsatorn [35]	NR	15 (1829)	NR	RR	7.30 (0.13, 414.04)	M	NMA conducted

*NA* = not applicable; *NMA* = network meta-analysis; *NR* = not reported; *OR* = odds ratio; *RR* = risk ratio; *RD* = risk difference; *H* = High, *M* = Moderate, *L* = Low, *CL* = Critically low; *RCT* = randomised controlled trial

Overall, most reviews showed no significant difference in recurrence rates between different mesh fixation techniques regardless of whether open or laparoscopic surgery was used.

## Discussion

In both open and laparoscopic inguinal hernia repairs, several techniques for mesh fixation have been studied in the attempt to keep the mesh in the appropriate position whilst reducing the pain experienced by patients after surgery and limiting the incidence of recurrence. A consensus regarding which technique is optimal has yet to be reached and, at present, the decision about which to use is often based on the surgeon’s preference. This overview examined evidence from 20 systematic reviews (one Cochrane systematic review and 19 non-Cochrane reviews) assessing methods for mesh fixation during both open and laparoscopic surgical approaches.

Regarding the rate of chronic pain after open mesh repair for inguinal hernia, the results of this overview show a consistent pattern in favour of glue fixation compared with suture fixation, whereas there was no clear indication of a significant difference between the self-gripping method and sutures. It is worth noting that the systematic review by Rausa et al. [24] was the only one assessing all three methods of mesh fixation (suture fixation, self-gripping mesh and glue fixation) using a network meta-analysis involving a total of 20 RCTs. The network meta-analysis results showed similar rates of chronic pain among the three methods of mesh fixation, with no significant increase of chronic pain after glue fixation or self-gripping mesh fixation. Glue fixation was ranked as the method with the highest probability of reducing the risk of chronic pain, followed by self-gripping and suture. Regarding the risk of recurrence, most reviews showed similar rates of recurrence between the assessed methods for mesh fixation. Although there was a trend in favour of suture compared with glue fixation, there was no clear evidence of an increased risk of recurrence after glue fixation. Similarly, there was no evidence that self-gripping mesh provided additional benefits compared with suture.

With regards to laparoscopic mesh repairs, due to the low number of studies available for each repair method, most reviews did not report results separately for TEPP and TAPP approaches or perform relevant sensitivity analyses. The results of this overview show that, in general, glue fixation (four systematic reviews) had a lower rate of chronic pain compared to mechanical fixation (tack and/or staple). The recent network meta-analysis by Techapongsatorn et al., assessed 11 RCTs and found no significant difference between various types of mesh fixation for laparoscopic hernia repair (i.e., suture, metallic tack, absorbable tack, glue, no fixation); however, glue was ranked the best for reducing chronic pain compared to suture and glue and suture were ranked the highest for lowering the incidence of recurrence compared to mechanical fixation. Little information was available from the identified systematic reviews to assess glue fixation versus self-gripping mesh.

In general, there is an indication that non-penetrating methods of mesh fixation such as glue fixation have a reduced risk of chronic pain compared to mechanical fixation and suture. This is in line with the notion that techniques, which can reduce tissue trauma and nerve injuries, are likely to reduce the risk of chronic pain. On the other hand, suture appears to increase the risk of chronic pain and there was only limited evidence that this could lower the risk of recurrence. While there is still a lack of robust evidence to support the routine use of glue fixation in open and laparoscopic hernia repairs, it may be effective in reducing chronic pain without increasing the risk of recurrence and, therefore, can be regarded as a suitable alternative to mechanical fixation or suture.

There are some limitations to the present overview. There were considerable differences in the inclusion and exclusion criteria among the included systematic reviews, such as the type of hernia (unilateral and bilateral inguinal hernias, primary and recurrent inguinal hernias), age of participants, and the duration of follow-up. In some systematic reviews, evidence was restricted to a small number of trials (some of them of small sample size, hence not powered to detect significant differences between treatment arms) or to a single trial.

There was also a considerable disparity across existing reviews in terms of the type of fixation techniques that were compared, and, in the way, postoperative pain was defined and measured. This was the consequence of the fact that all reviews included a pool of heterogeneous trials with mixed characteristics. For example, various types of glue (chemical and biological), mechanical fixation methods (staple, tack, metallic tack, and absorbable tack) and sutures (absorbable and non-absorbable), as well as various types of mesh (e.g., lightweight mesh, heavyweight mesh) were used across included studies. This hampered the comparability of the studies included in each review and therefore the reliability of the overall review findings. There was also a lack of clear subgroup analyses within the identified reviews, which could have been useful to investigate the heterogeneity between included studies. Almost all reviews reported outcomes (chronic pain and recurrence) within 12 months with only one systematic review reporting recurrence rates at 5 years [26]. Therefore, this limits the findings of this overview to ‘chronic pain’ that presents up to 1 year postoperatively and not pain thereafter. It is not clear whether mesh fixation methods will differ in long-term recurrence rates especially if we consider that the use of mesh in inguinal hernia repairs is associated with late rather than early recurrence and that new recurrences may present within 5 years post-surgery [39, 40]. Almost no information was provided on hernia size and the impact and limitations of using some fixation techniques for larger hernias (e.g., glue or self-gripping) are still unclear. Similarly, no information was reported on the expertise of the surgeons performing the mesh procedures.

Whilst some of the existing reviews recognised that some fixation techniques are more expensive than others (e.g., glue, self-gripping), none properly assessed the cost-effectiveness of routine use of the different techniques for mesh fixation in clinical practice. This overview focused exclusively on evidence published in full in English; we cannot exclude the likelihood of having missed some relevant publications available in other languages. We used the AMSTAR-2 tool to assess the method of the included systematic reviews [23], but we did not attempt to re-assess the risk of bias of the trials included in each systematic review. Furthermore, we did not quantify the degree of overlap between reviews in terms of included trials.

In conclusion, based on the results of the existing systematic reviews there is an indication that glue fixation may be more effective than other techniques in reducing the rate of chronic pain presenting up to 1 year postoperatively without increasing the risk of recurrence. However, current systematic reviews of RCTs assessing methods for mesh fixation after open or laparoscopic inguinal hernia repair have a variable degree of quality and considerable heterogeneity. We believe that there is no need for further systematic reviews on this clinical topic unless new RCTs are published or new techniques are developed. Future research should consider various methods of mesh fixation alongside the type of mesh (lightweight versus heavyweight mesh; synthetic versus biological meshes), the size and location of the hernia defect as well as the type of surgical approach chosen. There is a clear need for a uniform definition and assessment of postoperative pain in patients who undergo inguinal hernia repair as well as a need to fully understand the impact of different fixation techniques on clinical outcomes. To inform clinical practice and improve the burden that chronic pain and risk of recurrence pose to a significant number of patients, future research should assess longer-term outcomes (> 1 year), including patient’s quality of life, as well as the cost-effectiveness of different fixation techniques. It is worth noting that recurrent surgery is technically much more difficult than primary surgery and with higher risks of complications (including chronic pain). Therefore, establishing the optimal treatment of a primary inguinal hernia procedure to reduce recurrences would be extremely important to move hernia surgery forward.

## Data Availability

Not applicable.
